# Supplementary material for: Rege-1 promotes C. elegans survival by modulating IIS and TOR pathways
Source: PLoS Genet. 2023 Aug 9;19(8):e1010869. doi: 10.1371/journal.pgen.1010869 (PMC10441803; doi:10.1371/journal.pgen.1010869)
Supplement: S4 Table — (DOCX) [file pgen.1010869.s008.docx]

|  | ets-4(rrr16) vs WT (Pekec, Tina et al. Nat Commun2022) | | |
| --- | --- | --- | --- |
|  | ets-4(OK165) vs WT | rege-1(imm070) vs WT | rege-1(imm070); ets-4(ok165) vs WT |
| r | 0.2326 | -0.3218 | -0.297 |
| P (two-tailed) | 0.0002 | <0.0001 | <0.0001 |
| P value summary | *** | **** | **** |
| Number of XY Pairs | 253 | 253 | 1312 |

S4 Table: Comparison of Differentially Expressed Genes in This Study and Previously Published Datasets

|  | ets-4(ok165) vs WT (Thyagarajan et al. PLOS Genetics 2010) | | | |
| --- | --- | --- | --- | --- |
|  | OP50 | | PA14 | |
|  | rege-1(imm070) vs WT | rege-1(imm070); ets-4(ok165) vs WT | rege-1(imm070) vs WT | rege-1(imm070); ets-4(ok165) vs WT |
| r | -0.7485 | -0.9455 | -0.5708 | -0.8105 |
| P (two-tailed) | 0.0203 | <0.0001 | 0.0848 | <0.0001 |
| P value summary | * | **** | ns | **** |
| Number of XY Pairs | 9 | 21 | 10 | 23 |

|  | genes upregulated in rege-1 (rrr13) vs.N2 (Habacher et al, Dev. Cell 2016) | | | |
| --- | --- | --- | --- | --- |
|  | OP50 | | PA14 | |
|  | rege-1(imm070) vs WT | rege-1(imm070) vs rege-1(imm070);ets-4(ok165) | rege-1(imm070) vs WT | rege-1(imm070) vs rege-1(imm070);ets-4(ok165) |
| r | 0.749 | 0.5547 | 0.5193 | 0.5243 |
| P (two-tailed) | <0.0001 | <0.0001 | <0.0001 | <0.0001 |
| P value summary | **** | **** | **** | **** |
| Number of XY Pairs | 81 | 83 | 60 | 64 |

|  | genes downregulated in rege-1(rrr13);ets-4i vs.rege-1(rrr13) (Habacher et al, Dev. Cell 2016) | | | |
| --- | --- | --- | --- | --- |
|  | OP50 | | PA14 | |
|  | rege-1(imm070) vs WT | rege-1(imm070) vs rege-1(imm070);ets-4(ok165) | rege-1(imm070) vs WT | rege-1(imm070) vs rege-1(imm070);ets-4(ok165) |
| r | -0.7531 | -0.577 | -0.4731 | -0.4871 |
| P (two-tailed) | <0.0001 | <0.0001 | 0.0001 | <0.0001 |
| P value summary | **** | **** | *** | **** |
| Number of XY Pairs | 81 | 83 | 60 | 64 |
