# Supplementary material for: Rege-1 promotes C. elegans survival by modulating IIS and TOR pathways
Source: PLoS Genet. 2023 Aug 9;19(8):e1010869. doi: 10.1371/journal.pgen.1010869 (PMC10441803; doi:10.1371/journal.pgen.1010869)
Supplement: S6 Table — (DOCX) [file pgen.1010869.s010.docx]

| Gene Name | strand | Sequence(5’→3’) | Referance |
| --- | --- | --- | --- |
| act-3 | F | GGCCCAATCCAAGAGAGGTATCC | This study |
|  | R | GGGCAACACGAAGCTCATTGTA | This study |
| rege-1 | F | CGGCAAATGAATGTTTATCCAG | Habacher et al., 2016 |
|  | R | ATCAGATCCAGTATTCACAGGTC | Habacher et al., 2016 |
| ets-4 | F | CTGAGAACCCGAATCATCCA | Habacher et al., 2016 |
|  | R | TCATTCATGTCTTGACTGCTC | Habacher et al., 2016 |
| ins-7 | F | GCATGCGAATCGAATACTGAAG | This study |
|  | R | GAAGAAATTAAGGACAGCACTGTTT | This study |
| ech-9 | F | TGGAACAGATTATTCGTCGAGTT | This study |
|  | R | ACACCGGTAACCTTCATTTACA | This study |
| asp-12 | F | CGATGGAACATGCAAAGGAAAG | This study |
|  | R | TTAGCGCTTCCAGATTCGTATT | This study |
| spp-3 | F | TTCTCATCGACAGAGGCTAATG | This study |
|  | R | CGTCGACGGCCTTCTTAAT | This study |
| clec-52 | F | CTTTCCCAGCTGTTACAATTCC | This study |
|  | R | GAGCATCATTGAAATTGGTGTATTG | This study |
| acox-1.5 | F | TGTATACTTGCTCGGTTGGTG | This study |
|  | R | GGTGCCATTTCTTCTCCCTT | This study |

Table S6 primer list used in this study

Guide RNA and repair template used in this study for CRISPR:

|  | Sequence(5’→3’) |
| --- | --- |
| sgRNA for rol-6 | CGTGTGAGACGTCAACAATAG |
| Repair template for rol-6 | TGTGGGTTGATATGGTTAAACTTGGAGCAGGAACCGCTTCCAACCGTGTGCGCTGCCAACAATA |
| sgRNA for D231N | TTGAGAGCTGTGGTTGTTGA |
| Repair template for D231N | ACGTCATCATCCACACCATCCAAATACAACCCGGATCCAAGTTTGAGAGCCGTTGTAGT  AAATGGATCAAATGTTGCAATGTTGTAAGTTGACGCAAAACAAATGGTAGAAA |
