# Supplementary material for: Rege-1 promotes C. elegans survival by modulating IIS and TOR pathways
Source: PLoS Genet. 2023 Aug 9;19(8):e1010869. doi: 10.1371/journal.pgen.1010869 (PMC10441803; doi:10.1371/journal.pgen.1010869)
Supplement: Attachment — Submitted filename: Tsai PLOS Genetics comments.pdf [file pgen.1010869.s011.pdf]

Summary
Ribonucleases play important roles in rapid post-transcriptional control of gene expression by modulating mRNA stability, especially in response to environmental stimuli. Here Tsai and Tsai describe their studies of REGE-1, the *C. elegans* ortholog of mammalian Regnase-1, a ribonuclease with diverse cell-type specific functions in immunity as well as in tumor cells where it regulates programmed cell death. To functionally characterize REGE-1, the authors use CRISPR gene editing to create a new *rege-1* allele (*ht070*) bearing a missense mutation that presumably renders a ribonuclease-dead version of the protein. Compared to wildtype animals, *rege-1(ht070)* mutants are short-lived on *E. coli* and are more susceptible to infection by *Pseudomonas aeruginosa*. Both phenotypes appear to be attributable to *ets-4* whose mRNA is a known substrate of REGE-1. ETS-4 is an evolutionarily conserved transcription factor that the authors show is upregulated upon bacterial infection. Through RNAseq analysis, the authors find significant overlap between ETS-4 transcriptional targets and genes whose expression is negatively regulated when the FOXO transcription factor DAF-16 is activated in the absence of insulin signaling, a group referred to as Class 2 genes previously shown to limit *C. elegans* lifespan. Among putative ETS-4-regulated Class 2 genes are *ins-7* and *acox-1.5*, with connections to the insulin signaling (IIS) pathway and the target of rapamycin (TOR) pathway, respectively, both of which inhibit immunity in *C. elegans*. The *rege-1* enhanced susceptibility to pathogen phenotype is masked when combined with a loss of function mutation in the *daf-2* insulin receptor and is partially rescued by *ins-7*, suggesting that immunity in *rege-1 (ht070)* animals is diminished because of IIS activation. Supporting this possibility, quantitative gene expression and *in vivo* reporter analyses substantiate the RNAseq results and indicate that *ins-7* expression is positively regulated by ETS-4. Although genetic ablation of TOR signaling through a mutation in the GTPase activator *raga-1* completely rescues the *rege-1* immune deficiency, it is only partially rescued by knockdown of other purported upstream activators of TOR, including *acox-1.5*. Based on these data, the authors propose a model in which REGE-1 attenuates IIS signaling and TOR activity in response to environmental stimuli by degrading *ets-4* mRNA, a function that contributes to immunity during bacterial infections.

Overall Comments
The authors make several notable contributions to their field with this study. First, they have created a new allele of *rege-1* (named *ht070*), yielding a D231N substitution in the predicted active site of the protein that appears to impair its ability to degrade mRNA. Prior to this study, another group had generated a catalytic null version of *rege-1(rrr21)* that combined the D231N mutation with three other amino acid substitutions (D313A, D314A, D332A; Habacher *et al*., *Dev. Cell* 2016). Tsai and Tsai's functional characterization of *rege-1(ht070)* mutants suggests that the D231N mutation on its own may be sufficient to inhibit the enzymatic activity of REGE-1. Another advance that this study makes is that it provides evidence to reject a hypothesis derived from Habacher's study whereby the increased expression of ETS-4-regulated immunity genes in *rege-1* mutants may have been predicted to confer those animals with resistance to bacterial infection. Tsai and Tsai demonstrate that *rege-1* mutants are in fact more susceptible to *P. aeruginosa* infection, indicating that at high dosages, products of ETS-4-regulated genes may impair host defense or cause toxicity. Suppression of these genes by REGE-1 could therefore be consistent with the role of Regnase-1 in downregulating proinflammatory genes during the vertebrate immune response. Finally, Tsai and Tsai build upon the observation of overlap between genes regulated by ETS-4 and by the IIS pathway reported in a previous study (Thyagarajan *et al., PLOS Genetics*, 2010). Whereas Thyagarajan found only 5 genes to be regulated by both IIS and ETS-4, the current study uncovered at least 41 genes that are

downstream of DAF-16 (Class 2 genes) to also be potential transcriptional targets of ETS-4. This helps to explain the genetic interaction between *daf-16* and *ets-4*, and it raises the possibility of dynamic regulation of Class 2 genes by DAF-16 and ETS-4. Interestingly, the authors provide several lines of evidence to suggest that ETS-4 stimulates IIS through upregulation of *ins-7*, encoding an agonist of the DAF-2 insulin receptor (Fig. 3). For example, in *rege-1* mutants where ETS-4 levels are elevated *ins-7* expression is 4-5-fold higher than in wildtype animals. Loss of *ins-7* function partially rescues the pathogen susceptibility phenotype of *rege-1* mutants, as would be expected if IIS-mediated inhibition of DAF-16 is relieved. Together these data indicate that the immunodeficiency of *rege-1* mutants is due in part to sustained Class 2 gene expression through ETS-4-mediated propagation of insulin signaling to block transcriptional activity of DAF-16.

Despite the strengths of the study, there are important issues that need to be addressed in order to solidify the authors' conclusions and support their proposed model (Fig. 4E). These include the following: 1) quality and analysis of RNASeq data, 2) verifying the connection between *acox-1.5, ech-9*, and TOR signaling, 3) distinguishing ETS-4 from PQM-1, and 4) reassessing the interpretation of some pieces of data or how the interpretation is presented. In addition, the content of the Introduction and Discussion sections should be significantly revised, and there are a substantial number of errors in spelling and formatting throughout the manuscript.

Major Concerns

Differential Gene Expression (DGE) studies

1. Statistical analyses of the DGE dataset should be more forthcoming and should be included in the supplement. At a minimum the authors should include volcano plots correlating changes in gene expression between pairs of samples with their statistical significance as well as principal component analyses to demonstrate clustering among their samples.

2. In the absence of principal component analysis, the only way for a reader to assess the reproducibility of the authors' replicates is to compare gene expression patterns of replicate samples of the same genotype in the heatmap in Fig. 1E. While only a rough estimate, this type of analysis indicates that the expression patterns between replicates appear to be largely consistent with one possible exception: the two PA14-fed *ets-4* samples. The expression patterns of these samples seem dissimilar in many cases, and especially for genes that are upregulated in response to PA14 in the absence of *rege-1* (bottom ~1/6th of the heat map). Whether or not there is a true difference between these samples depends on the results of statistical analyses, but in any case the authors should strongly consider performing RNAseq with at least one additional replicate of RNA from PA14-fed *ets-4* mutants and incorporating the results into new DGE analysis.

3. The authors could more clearly describe the categories of genes potentially regulated by ETS-4 that are revealed in their DGE analyses (heat map in Fig. 1E). In particular, there seem to be 6 categories: 1) positively regulated by ETS-4 on both OP50 and PA14, 2) positively regulated by ETS-4 on OP50, 3) positively regulated by ETS-4 on PA14, 4) negatively regulated by ETS-4 on both OP50 and PA14, 5) negatively regulated by ETS-4 on OP50, and 6) negatively regulated by ETS-4 on PA14.

- Lists of genes in each category should be provided in supplemental material
- Enriched GO terms (with statistics) should be provided for each category
- Where do Class 2 genes fall in these categories?

- The authors should comment on the role of ETS-4 as a transcriptional repressor in addition to its role as a transcriptional activator.

4. Along the lines of the comment above, a comprehensive list of differentially regulated genes needs to be made available to readers. The authors should include a link to the complete dataset from their RNAseq/DGE studies on NCBI's Gene Expression Omnibus (GEO) website.

5. For genes predicted to be regulated by ETS-4, the authors should use Wormbase tools to perform enrichment analyses for GO terms, phenotypes, and tissue expression patterns.

6. As they did for *ins-7* in Fig. 3A, the authors should use qRT-PCR to validate the results of their RNAseq data regarding the expression levels of the five putative ETS-4 transcriptional targets (*acox-1.5*, *asp-12, clec-52, ech-9,* and *spp3*).

Connection between ETS-4 and the TOR pathway

7. Regulation of TOR by ETS-4 and the consequent effect on immunity is a major tenet of the authors' model. The authors' strongest evidence of a potential connection between ETS-4 and the TOR pathway are genetic data suggesting an epistatic relationship between *rege-1* and *raga-1*, encoding a TORC1 interactor (Fig. 2C). Other data purportedly linking ETS-4 and TOR are less clear.

The authors' first indication that ETS-4 may intersect with the TOR pathway was the observation of overlap between their DGE dataset of genes upregulated in *rege-1* mutants and genes that are downregulated in *rsks-1* mutants. *rsks-1* encodes an ortholog of S6 kinase, a ribosomal protein that is directly phosphorylated by mTORC1 in mammals, and genetic data implicate that *rsks-1* as a component of the TOR pathway in *C. elegans*. Surprisingly, the authors' RNASeq data does not indicate that there is any differential expression of "core components, substrates, interactors or regulators" of TOR signaling, raising the question of what specific genes in the authors' DGE dataset were identified by the WormExp database as being in common with those downregulated in the absence of *rsks-1*. Do these genes really point to a change in TOR activity in *rege-1* animals? Alternatively, could there be a similar compensatory response at the level of gene expression (independent of TOR) to mutations that increase mRNA stability (*rege-1*) and reduce the rate of protein translation (*rsks-1*)—perhaps upregulation of ribosomal biogenesis? In any case, as they did for *ins-7* in Fig. 3B, the authors should validate the results of their RNAseq data by using qRT-PCR to confirm differences in expression for genes they believe to be related to the TOR pathway.

8. The authors claim that two ETS-4-regulated genes, *acox-1.5* and *ech-9*, whose products function sequentially in the peroxisomal beta oxidation of fatty acids, regulate TOR in *C. elegans*. To implicate *acox-1.5* as a potential upstream activator of TOR, the authors cite a study pertaining to the murine ortholog Acox1 that appears to have such a function in the liver (He *et al., Mol. Cell* 2020). Since a direct link between *acox-1.5, ech-9* and TOR in *C. elegans* has not yet been reported in the literature, the authors need to produce evidence in support of this possibility themselves. One possibility would be to make use of a fluorescent reporter of TOR activity such as LGG-1::RFP, which allows for relative TOR activity to be measured based on quantification of autophagic vesicles within intestinal cells (Schieber and Chandel, *Cell Rep.* 2014). The authors could ask whether intestinal cells in *acox-1.5* and *ech-9* mutants have more autophagic vesicles than wildtype animals, indicating reduced TOR activity. It would also be

prudent to use this assay to measure TOR activity in *rege-1* mutants to verify that it is enhanced upon increased ETS-4 activity.

9. The genetic data that the authors report regarding *raga-1, acox-1.5*, and *ech-9* do not convincingly indicate that these genes necessarily function in the same pathway. If *ech-9* is downstream of *acox-1.5* in the same biosynthetic pathway that contributes to the production fatty acid beta-oxidation metabolites to activate TOR as the authors suggest, then mutations in *ech-9, acox-1.5* and other genes that positively regulate the TOR pathway would be expected to yield similar phenotypes. However, this is not what the authors found. While *ech-9* mutations have little if any effect on the survival of *P. aeruginosa*-infected *C. elegans* (Fig. 4C), *acox-1.5* mutations cause animals to be more susceptible to bacterial infection (Fig. 4A). On the other hand, mutations in *raga-1* encoding a Rag GTPase that activates TOR, appear to make animals slightly more resistant to bacterial infection (Fig. 2C). Can the authors offer an explanation for these results?

Comparison of ETS-4 to PQM-1

10. The prevailing model regarding the regulation of gene expression by the IIS pathway is that DAF-16 functions as a transcriptional activator of Class 1 genes while PQM-1 upregulates Class 2 genes. Nuclear localization of the two transcription factors appears to be mutually exclusive such that in *daf-2* animals where DAF-16 is constitutively active, PQM-1 remains in the cytosol, unable to access its transcriptional targets. Considering that ETS-4 also regulates Class 2 genes, how does it fit into this regulatory scheme?

The authors state that out of a total of 58 Class 2 genes that appear to be upregulated in an ETS-4-dependent manner, 49 are direct transcriptional targets of PQM-1. This may suggest that the two transcription factors co-regulate genes, perhaps in a cooperative manner, or that they are functionally redundant. Despite the apparent similarities between ETS-4 and PQM-1, there are indications from previous studies that they have distinct roles. For example, the lifespan of *pqm-1* mutants is comparable to that of wildtype animals (Tepper *et al., Cell* 2013), but *ets-4* mutants are long-lived (Thyagarajan *et al*., *PLOS Genetics*, 2010). Further, while *pqm-1* is required for lifespan extension when IIS is inhibited, *ets-4* is not. The authors have an opportunity in the current study to further distinguish ETS-4 from PQM-1 through experiments that directly compare them to each other. In particular, they should consider addressing the following questions:

- What is the total extent of overlap between genes that seem to be regulated by both ETS-4 and PQM-1? That is, in addition to Class 2 genes, how many other genes that the authors believe to be regulated by ETS-4 have been identified as direct PQM-1 transcriptional targets?
- Is the *rege-1* pathogen susceptibility phenotype rescued by mutations in *pqm-1* (or *pqm-1* knockdown) as it is by mutations in *ets-4*?
- Does a loss-of-function of *pqm-1* rescue the shortened lifespan of *rege-1* mutants?
- Is the nuclear localization of ETS-4 and DAF-16 mutually exclusive, as is the case for PQM-1 and DAF-16? An *ets-4::gfp* strain already exists (Habacher, *Dev. Cell* 2016), and the authors could examine its subcellular localization in presence and absence of DAF-16.

Data interpretation

11. In at least two cases the authors make statements in the Results section that appear to be at odds with what the data show. These instances are as follows:

1) In their description of the oil red O staining in Fig. 2D, the authors state that manipulation of either the IIS or TORC1 signaling pathways "is insufficient to rescue the fat loss phenotype of *rege-1 (ht070)"* (lines 207-208). However, the quantitation of staining in Fig. 2E shows that *rege-1;daf-2* animals stained more intensely than both *rege-1* and wildtype animals. The authors should reevaluate their assessment of the data and should also perform additional statistical tests to examine the differences between *rege-1;daf-2* and *rege-1* mutants in particular.

2) In the Abstract, the authors claim that knocking down *acox-1.5* "completely abrogated the survival difference between *rege-1* mutants and wildtypes [*sic*]". This claim is repeated again in the Results section (lines 243-244) and Discussion section (lines 275-276), but it is not what the data show. RNAi targeting *acox-1.5* impairs immunity in both wildtype animals and *rege-1* mutants. Combining the *acox-1.5* knockdown with the *rege-1* loss of function mutation reduces the survival of infected animals to the same extent as does *acox-1.5* knockdown alone. It does not rescue the enhanced susceptibility to pathogen of *rege-1* mutants or restore their survival back to wildtype levels. As written, the authors' description of the data is unclear and potentially misleading.

12. While statistically significant, the magnitude of the difference in mean lifespan in *rege-1* strains where ETS-4-regulated Class 2 genes is knocked down by RNAi is relatively low and is even lower in *rege-1;ins-7* double mutants (Fig. 4C and D). Is there a threshold for a fold change in mean or maximum lifespan (or some other metric) that the authors established to determine whether or not a specific RNAi treatment had a substantial impact on the survival of *rege-1* animals? Or was statistical significance the only criterion?

Comments on Specific Figures

1. Fig. 1B shows that *rege-1* expression is elevated in the *rege-1(ht070)* mutants as compared to wildtype. The authors may wish to point out that this makes the increased *ets-4* expression in that strain even more remarkable and that it is a strong indication that the gene product of the *ht070* allele has little to no ribonuclease activity.

2. Regarding Fig. 2:

- Fig. 2A should not be included as a figure panel but rather should be converted into a separate table.
- callout for Fig. 2B precedes the callout for Fig 2A in the text.
- In the figure legend Panels B and C are not described in sufficient detail. The authors need to mention all genotypes tested.

3. In Fig. 4 panels C and D the color scheme in the symbol key should match that in the plot—specifically, the shade of green used to present RNAi-treated *rege-1* mutants in the key is not the same shade as in the plots.

4. Regarding Fig S1:

- To avoid the first call-out in the text being to a supplemental figure (Fig. S1) could at least some of the data in that figure be moved to Fig. 1?

- What statistical significance is indicated by the asterisks in the symbol key? (Mention p-value but no specific number)

5. The functional characterization of *asp-12* seems to be out of place in Fig. S2, since the other panels in that figure pertain to read counts for TOR pathway components. Regarding the *asp-12* knockdown data:
   - The authors could consider including it as part of Fig. 4, but for the sake of completeness this would require additional experiments to knockdown *asp-12* in wildtype and *rege-1* mutants to generate the same type of data as shown for *ech-9* and *clec-52* in panel C.
   - Why was *spp-3* the one ETS-4 regulated gene that was excluded from functional analyses?

6. Regarding Table S1:
   - consider including average values from all replicates of a particular experiment to reflect the data that are depicted in corresponding figure panels
   - consider breaking this up into separate supplemental tables to accompany specific figures instead of compiling all statistical data in one table. For example, the statistical data supporting Fig S1B and especially S1C (PA14 infection) could be moved up to appear immediately after the data pertaining to Fig. 1 so that the reader can directly compare the behavior of the two *rege-1* mutant strains (*tm2265* deletion strain and presumptive ribonuclease-dead *ht070*)
   - the statistical analysis of lifespan date should include p-values, as was provided for data pertaining to survival curves in Fig S1B and S1C but not for others.
   - the units of the values in the “mean lifespan” column are presumably days, but this should be specified. Also, is this truly mean lifespan or is it median lifespan (LT50)?

Comments on Writing

Introduction

1. The introduction should be reorganized to first provide the reader with background information on post-transcriptional gene regulation and ribonucleases in particular.

2. The authors should add a paragraph discussing how mRNA degradation by ribonucleases allows animals to dynamically respond to their environment, in part by regulating metabolic pathways.

3. Comment on the significance of the research question, as defined on lines 77-79.

Methods

4. More details pertaining to CRISPR gene editing methodology should be included. In particular, the authors should cite or describe the protocol that they followed and provide specific information regarding reagents, including guide RNA, repair templates, and the sequences of genotyping primers.

5. Provide an explanation of the technique used to quantify GFP fluorescence intensity for the micrographs in Fig. 3C and for the quantification of oil red O staining in Figs. 2D and 4B. A description of statistical analyses should also be included.

6. Explain how animals were synchronized for RNAseq experiments and for oil red O staining experiments.

Discussion
7. Although it appears under the subheading of "Model," the first paragraph reiterates the summary of results that appears at the end of the Introduction section. It should be modified to describe the authors' model in Fig. 4E.

8. The authors need to do significantly more to place their work in the context of other studies in the literature. At a minimum, they should specifically compare their findings to the following three papers:

1) Thyagarajan *et al. PLOS Genetics* 2010 (ETS-4)
2) Habacher *et al, Dev. Cell* 2016 (REGE-1)
3) Tepper *et al., Cell* 2013 (PQM-1)

9. Based on their GO term analyses of their DGE data, can the authors speculate as to why increased expression of ETS-4-regulated genes shortens lifespan and reduces immunity? Is there something in common to these genes that would make them particularly detrimental when expressed at high levels?

10. There is not a sufficient explanation of how fat and fat metabolism are related to immunity. This issue is pertinent to this study since the authors test for rescue of the *rege-1* fat storage defect by inhibition of insulin signaling and TOR activity. The authors should comment on that topic in the Discussion and reference papers such as Anderson *et al., PLOS Pathogens* (2019).

11. Consider addressing the evolutionary basis of a pathway (REGE-1-mediated mRNA degradation) that potentially came about in order to limit the host's response to infection (upregulation of ETS-4 transcriptional targets). How does this compare with other pathways that are induced by environmental stimuli? Are there similar regulatory mechanisms for responses unrelated to host defense in "lower" organisms?

Spelling, grammar, and formatting
12. There are many spelling and grammar errors throughout the manuscript. In addition, the authors need to be careful to use the proper *C. elegans* conventions for the formatting of gene and protein names.

13. Lines 158 and 159: need citations for Class 1 and Class 2 genes as well as CHIP data for DAF-16 and PQM-1 referred to in the text

14. Line 277: Why is there a callout to Fig. 4E when the sentence mentions a mouse study?
