# Supplementary material for: Rege-1 promotes C. elegans survival by modulating IIS and TOR pathways
Source: PLoS Genet. 2023 Aug 9;19(8):e1010869. doi: 10.1371/journal.pgen.1010869 (PMC10441803; doi:10.1371/journal.pgen.1010869)
Supplement: Attachment — Submitted filename: Tsai PLOS Genetics comments.pdf [file pgen.1010869.s012.pdf]

## Reviwer 1:

## Summary

Ribonucleases play important roles in rapid post-transcriptional control of gene expression by modulating mRNA stability, especially in response to environmental stimuli. Here Tsai and Tsai describe their studies of REGE-1, the *C. elegans* ortholog of mammalian Regnase-1, a ribonuclease with diverse cell-type specific functions in immunity as well as in tumor cells where it regulates programmed cell death. To functionally characterize REGE-1, the authors use CRISPR gene editing to create a new *rege-1* allele (*ht070*) bearing a missense mutation that presumably renders a ribonuclease-dead version of the protein. Compared to wildtype animals, *rege-1(ht070)* mutants are short-lived on *E. coli* and are more susceptible to infection by *Pseudomonas aeruginosa*. Both phenotypes appear to be attributable to *ets-4* whose mRNA is a known substrate of REGE-1. ETS-4 is an evolutionarily conserved transcription factor that the authors show is upregulated upon bacterial infection. Through RNAseq analysis, the authors find significant overlap between ETS-4 transcriptional targets and genes whose expression is negatively regulated when the FOXO transcription factor DAF-16 is activated in the absence of insulin signaling, a group referred to as Class 2 genes previously shown to limit *C. elegans* lifespan. Among putative ETS-4-regulated Class 2 genes are *ins-7* and *acox-1.5*, with connections to the insulin signaling (IIS) pathway and the target of rapamycin (TOR) pathway, respectively, both of which inhibit immunity in *C. elegans*. The *rege-1* enhanced susceptibility to pathogen phenotype is masked when combined with a loss of function mutation in the *daf-2* insulin receptor and is partially rescued by *ins-7*, suggesting that immunity in *rege-1 (ht070)* animals is diminished because of IIS activation. Supporting this possibility, quantitative gene expression and *in vivo* reporter analyses substantiate the RNAseq results and indicate that *ins-7* expression is positively regulated by ETS-4. Although genetic ablation of TOR signaling through a mutation in the GTPase activator raga-1 completely rescues the rege-1 immune deficiency, it is only partially rescued by knockdown of other purported upstream activators of TOR, including acox-1.5. Based on these data, the authors propose a model in which REGE-1 attenuates IIS signaling and TOR activity in response to environmental stimuli by degrading *ets-4* mRNA, a function that contributes to immunity during bacterial infections.

## Overall Comments

The authors make several notable contributions to their field with this study. First, they have created a new allele of *rege-1* (named *ht070*), yielding a D231N substitution in the predicted active site of the protein that appears to impair its ability to degrade mRNA. Prior to this study, another group had generated a catalytic null version of *rege-1(rrr21)* that combined the D231N mutation with three other amino acid substitutions (D313A, D314A, D332A; Habacher *et al.*, *Dev. Cell* 2016). Tsai and Tsai's functional characterization of *rege-1(ht070)* mutants suggests that the D231N mutation on its own may be sufficient to inhibit the enzymatic activity of REGE-1. Another advance that this study makes is that it provides evidence to reject a hypothesis derived from Habacher's study whereby the increased expression of ETS-4-regulated immunity genes in *rege-1* mutants may have been predicted to confer those animals with resistance to bacterial infection. Tsai and Tsai demonstrate that *rege-1* mutants are in fact more susceptible to *P. aeruginosa* infection, indicating that at high dosages, products of ETS-4-regulated genes may impair host defense or cause toxicity. Suppression of these genes by REGE-1 could therefore be consistent with the role of Regnase-1 in downregulating proinflammatory genes during the vertebrate immune response. Finally, Tsai and Tsai build upon the observation of overlap between genes regulated by ETS-4 and by the IIS pathway reported in a previous study

(Thyagarajan *et al., PLOS Genetics*, 2010). Whereas Thyagarajan found only 5 genes to be regulated by both IIS and ETS-4, the current study uncovered at least 41 genes that are downstream of DAF-16 (Class 2 genes) to also be potential transcriptional targets of ETS-4. This helps to explain the genetic interaction between daf-16 and ets-4, and it raises the possibility of dynamic regulation of Class 2 genes by DAF-16 and ETS-4. Interestingly, the authors provide several lines of evidence to suggest that ETS-4 stimulates IIS through upregulation of ins-7, encoding an agonist of the DAF-2 insulin receptor (Fig. 3). For example, in rege-1 mutants where ETS-4 levels are elevated ins-7 expression is 4-5-fold higher than in wildtype animals. Loss of ins-7 function partially rescues the pathogen susceptibility phenotype of rege-1 mutants, as would be expected if IIS-mediated inhibition of DAF-16 is relieved. Together these data indicate that the immunodeficiency of rege-1 mutants is due in part to sustained Class 2 gene expression through ETS-4-mediated propagation of insulin signaling to block transcriptional activity of DAF-16.
Despite the strengths of the study, there are important issues that need to be addressed in order to solidify the authors' conclusions and support their proposed model (Fig. 4E). These include the following: 1) quality and analysis of RNASeq data, 2) verifying the connection between acox-1.5, ech-9, and TOR signaling, 3) distinguishing ETS-4 from PQM-1, and 4) reassessing the interpretation of some pieces of data or how the interpretation is presented. In addition, the Introduction and Discussion sections should be significantly revised, and there are a substantial number of errors in spelling and formatting throughout the manuscript.

## Major Concerns

### Differential Gene Expression (DGE) studies

1. Statistical analyses of the DGE dataset should be more forthcoming and should be included in the supplement. At a minimum the authors should include volcano plots correlating changes in gene expression between pairs of samples with their statistical significance as well as principal component analyses to demonstrate clustering among their samples.
   Thank you for your suggestions. We have included the principal component analysis of mRNAseq data of N2, *rege-1(imm070)*, and *rege-1(imm070);ets-4(ok165)* fed with either OP50 or PA14 in Figure S2B. After discussing with staff from WormBase, we have changed our strain name from *rege-1(hy070)* to *rege-1(imm070)*. We apologize for any confusion this may have caused.
   Additionally, we have removed the *ets-4(ok165)* data since the quality of the data for *ets-4(ok165)* fed on PA14 is not sufficient. Since the phenotype between N2, *ets-4(ok165)*, and *rege-1(imm070);ets-4(ok165)* are identical when fed with PA14. Therefore, we believe that two phenotypic wild-type controls (N2 and *rege-1(imm070);ets-4(ok165)*) are sufficient to help us narrow down the targets.
   As you suggested, we have provided the volcano plots and fold changes of all the datasets mention in the text in Supplementary Figure 2C and Supplementary Table 2, respectively.

2. In the absence of principal component analysis, the only way for a reader to assess the reproducibility of the authors' replicates is to compare gene expression patterns of replicate samples of the same genotype in the heatmap in Fig. 1E. While only a rough estimate, this type of analysis indicates that the expression patterns between replicates appear to be largely consistent with one possible exception: the two PA14-fed ets-4 samples. The expression patterns of these samples seem dissimilar in many cases, and especially for genes that are upregulated in response to PA14 in the absence of rege-1 (bottom ~1/6th of the heat map). Whether or not there is a true difference between these samples depends on

the results of statistical analyses, but in any case the authors should strongly consider performing RNAseq with at least one additional replicate of RNA from PA14-fed ets-4 mutants and incorporating the results into new DGE analysis.
Thank you for your suggestion. As you pointed out, the repeats of PA14-fed ets-4 samples have the largest differences between repeats compared to all other samples. Since we already have N2 and *rege-1(imm070);ets-4(ok165)* as phenotypic wild-type controls and PA14-fed ets-4 samples serve as an additional control, we have decided to exclude this sample from our analysis.

3. The authors could more clearly describe the categories of genes potentially regulated by ETS- 4 that are revealed in their DGE analyses (heat map in Fig. 1E). In particular, there seem to be 6 categories: 1) positively regulated by ETS-4 on both OP50 and PA14, 2) positively regulated by ETS-4 on OP50, 3) positively regulated by ETS-4 on PA14, 4) negatively regulated by ETS-4 on both OP50 and PA14, 5) negatively regulated by ETS-4 on OP50, and 6) negatively regulated by ETS-4 on PA14.
   - Lists of genes in each category should be provided in supplemental material
   - Enriched GO terms (with statistics) should be provided for each category
   - Where do Class 2 genes fall in these categories?
   - The authors should comment on the role of ETS-4 as a transcriptional repressor in addition to its role as a transcriptional activator.

Thank you for your suggestions. We have re-plotted the heatmap and separated it into six categories, as shown in Figure 2A. The list of genes in each category can be found in Table S2.
Although there is not much information from performing the Enrichment analysis, we have summarized our findings and included all the Enrichment analysis in Figure S2D. Moreover, we have provided evidence from the literature, such as Thyagarajan et al., (2010) in PLOS Genetics, and Li et al., (2015) in PLOS Genetics, which show that ets-4 indeed acts as a transcription activator.

4. Along the lines of the comment above, a comprehensive list of differentially regulated genes needs to be made available to readers. The authors should include a link to the complete dataset from their RNAseq/DGE studies on NCBI's Gene Expression Omnibus (GEO) website.

Thank you for your suggestion and we are terribly sorry that we forget to include the number at the first draft. We did upload the original raw data to NCBI's Gene Expression Omnibus (GEO) website, with the accession number GSE218235, we also provided the e-mail from GEO as prove. Now the data is available for reviewers, and the security token is "mlgbsseajvmjrot"

5. For genes predicted to be regulated by ETS-4, the authors should use Wormbase tools to perform enrichment analyses for GO terms, phenotypes, and tissue expression patterns.
Thank you for your suggestion. We have now included the enrichment analysis of genes predicted to be regulated by *ets-4* (*rege-1(imm070)* negatively regulated genes) in Figure S2D.

6. As they did for ins-7 in Fig. 3A, the authors should use qRT-PCR to validate the results of

their RNAseq data regarding the expression levels of the five putative ETS-4 transcriptional targets (acox-1.5, asp-12, clec-52, ech-9, and spp3).
Thank you for your suggestion. Though a slight rearrangement of the main text due to newly identify the ETS-4 ChIP seq data from ENCODE project, the mRNA quantities for all five gene mentioned above have been shown in either Figure 5A or Figure S4.

**Connection between ETS-4 and the TOR pathway**

7. Regulation of TOR by ETS-4 and the consequent effect on immunity is a major tenet of the authors' model. The authors' strongest evidence of a potential connection between ETS-4 and the TOR pathway are genetic data suggesting an epistatic relationship between rege-1 and raga-1, encoding a TORC1 interactor (Fig. 2C). Other data purportedly linking ETS-4 and TOR are less clear.

The authors' first indication that ETS-4 may intersect with the TOR pathway was the observation of overlap between their DGE dataset of genes upregulated in rege-1 mutants and genes that are downregulated in rsks-1 mutants. rsks-1 encodes an ortholog of S6 kinase, a ribosomal protein that is directly phosphorylated by mTORC1 in mammals, and genetic data implicate that rsks-1 as a component of the TOR pathway in C. elegans. Surprisingly, the authors' RNASeq data does not indicate that there is any differential expression of "core components, substrates, interactors or regulators" of TOR signaling, raising the question of what specific genes in the authors' DGE dataset were identified by the WormExp database as being in common with those downregulated in the absence of rsks-1. Do these genes really point to a change in TOR activity in rege-1 animals? Alternatively, could there be a similar compensatory response at the level of gene expression (independent of TOR) to mutations that increase mRNA stability (rege-1) and reduce the rate of protein translation (rsks-1)—perhaps upregulation of ribosomal biogenesis? In any case, as they did for ins-7 in Fig. 3B, the authors should validate the results of their RNAseq data by using qRT-PCR to confirm differences in expression for genes they believe to be related to the TOR pathway.

Thank you for your suggestion. We were indeed very intrigued by the discovery that no core protein in the mTORC1 signaling pathway was found to be up-regulated in the rege-1 mutant. In this revised paper, we have included several experiments to demonstrate the involvement of the rege-1 mutant in the poor PA14 survival phenotype associated with the activation of the TORC1 signaling pathway.

1. Knocking down two TOCR1 signaling pathway genes is sufficient to fully rescue the poor PA14 survival phenotype of rege-1 mutant.
   In addition to the raga-1 mutant as described previously, we have also used rsks-1 mutant to combine to *rege-1(imm070)*. Encouragingly, knocking down either of these essential TORC1 signaling pathway genes leads to full rescue of poor PA14 survival phenotype of *rege-1(imm070)* (Figure 4A-B)
2. Autophagy is inhibited in *rege-1(imm070)*.
   As you mentioned in one of your suggestions, it is well known that the activation of TORC1 signaling results in the inhibition of autophagosome formation, which we also briefly mentioned in the introduction. Here, we have used the *gfp::lgg-1* reporter in either wild-type and *rege-1(imm070)* strains to observed the autophagosome formation. To better visualize the differences of autophagosome formation, we have used *rab-7* RNAi to inhibits autophagosome and lysosome fusion (Figure 4C).

3. ETS-4 is not enriched among *rege-1* negatively regulated genes that are found down regulated in *daf-2;rsks-1* double mutant.
   As we mentioned in the previous version of manuscript, we did not observe significant changes in mRNAs of the C. elegans ortholog of core TORC1 pathway genes. To be sure whether the up-regulated genes found in *daf-2, rsks-1* are potential targets for ETS-4, we also identify the ETS-4 targets in the genes among the related datasets. Unlike the *rege-1* negatively regulated genes, which are significantly targeted by ETS-4, there is no significant enrichment of ETS-4 targeted genes in the overlap between rege-1 negatively regulated genes and the down-regulated genes in *rsks-1; daf-2*. Thus, it is reasonable to suspect that the increases in the TORC1 signaling pathway do not result from changes in mRNA but likely occur through post-translational modification.
4. The *acox-1.5* mutant largely rescued the poor PA14 survival of *rege-1(imm070)* and partially rescued the fat loss phenotype.
   Increased acetyl-CoA levels are known to promote TORC1 signaling, and fatty acid beta oxidation is one of the major source of acetyl-CoA. *Acox-1.5* is a key enzyme in the peroxisomal beta oxidation pathway and, despite the presence of six similar paralog proteins, our results in the revised manuscript showed that the acox-1.5 mutant resulted in fat accumulation and partially rescued the fat loss phenotype of *rege-1(imm070)*, suggesting that *acox-1.5* actively participates in the fatty acid beta oxidation pathway and likely contributes to the acetyl-CoA levels. Interestingly, the same mutant resulted in a 60% rescue of the poor PA14 survival phenotype in *rege-1(imm070)*, whereas all the single ETS-4 targeted genes tested were below 50%, suggesting that up-regulated acox-1.5 in *rege-1(imm070)* plays a major role in activating the TORC1 signaling pathway.

8. The authors claim that two ETS-4-regulated genes, acox-1.5 and ech-9, whose products function sequentially in the peroxisomal beta oxidation of fatty acids, regulate TOR in C. elegans. To implicate acox-1.5 as a potential upstream activator of TOR, the authors cite a study pertaining to the murine ortholog Acox1 that appears to have such a function in the liver (He et al., Mol. Cell 2020). Since a direct link between acox-1.5, ech-9 and TOR in C. elegans has not yet been reported in the literature, the authors need to produce evidence in support of this possibility themselves. One possibility would be to make use of a fluorescent reporter of TOR activity such as LGG-1::RFP, which allows for relative TOR activity to be measured based on quantification of autophagic vesicles within intestinal cells (Schieber and Chandel, Cell Rep. 2014). The authors could ask whether intestinal cells in acox-1.5 and ech-9 mutants have more autophagic vesicles than wildtype animals, indicating reduced TOR activity. It would also be prudent to use this assay to measure TOR activity in rege-1 mutants to verify that it is enhanced upon increased ETS-4 activity.

   Thank you for your suggestion. Although we were able to show that autophagy is decreased in the *rege-1(imm070)* background and that the use of the *acox-1.5(tm15936)* strain resulted in a cleaner and better rescue compared to RNAi, due to limited time for revision, we were unable to incorporate the double mutant into the *gfp::lgg-1* reporter construct. As mentioned by the reviewer, neither *acox-1.5* nor *ech-9* has been extensively studied. Considering the presence of six *acox-1* paralog proteins in C. elegans, it is difficult to determine whether *ech-9* is the direct downstream of *acox-1.5*. However, the new *acox-1.5* mutant clearly demonstrates its importance in fatty acid turnover, and thus, it is reasonable to believe that it plays an important role in altering acetyl-CoA levels, which in

turn affects raptor acetylation in TORC1. Therefore, we believe that this evidence is sufficient to highlight the importance of *acox-1.5* in the activation of TORC1 signaling. It is worth mentioning that although we did not observe any difference in mean lifespan between N2 and *rege-1(imm070)* after knocking down *acox-1* (as described in more detail in the results), which suggest the factor in *rege-1(imm070)* that cause the decreases in lifespan is blunted after knocking down *acox-1*, the *rege-1(imm070);acox-1.5(tm15936)* double mutant did not result in complete rescue of poor PA14 survival, suggesting that other differentially expressed genes may also contribute to the activation of TORC1, possibly including *ech-9*.

9. The genetic data that the authors report regarding raga-1, acox-1.5, and ech-9 do not convincingly indicate that these genes necessarily function in the same pathway. If ech-9 is downstream of acox-1.5 in the same biosynthetic pathway that contributes to the production fatty acid beta-oxidation metabolites to activate TOR as the authors suggest, then mutations in ech-9, acox-1.5 and other genes that positively regulate the TOR pathway would be expected to yield similar phenotypes. However, this is not what the authors found. While ech-9 mutations have little if any effect on the survival of P. aeruginosa-infected C. elegans (Fig. 4C), acox-1.5 mutations cause animals to be more susceptible to bacterial infection (Fig. 4A). On the other hand, mutations in raga-1 encoding a Rag GTPase that activates TOR, appear to make animals slightly more resistant to bacterial infection (Fig. 2C). Can the authors offer an explanation for these results?

Thank you for your feedback. In the revised paper, we have included a detailed analysis of the acox-1 and ech paralogs, including their mRNA and protein similarity. This provides a clear explanation for why knocking down acox-1.5 resulted in a global decrease in the survival of the rege-1 mutant and also there was little rescue found in ech-9 knockdown. Briefly, acyl-CoA oxidase 1 (ACOX1) and enoyl-CoA hydratase (ECH) are two contiguous steps in the peroxisomal beta-oxidation pathway. Six ACOX-1 paralogs and three ECH paralogs are found in the peroxisome. When using RNAi to knock down acox-1.5, all of the acox-1 paralogs showed 9% to 51% mRNA overlap with the acox-1.5 RNAi clone sequence, suggesting all of the paralog are likely been knockdown all at once. Therefore, knocking down acox-1.5 in the wild-type would be equivalent to knocking down all six acox-1 paralogs. For an unknown reason, this resulted in a decrease in C. elegans survival on PA14. However, observing no further decrease in PA14 survival after knocking down *acox-1* in *rege-1(imm070)* suggests that knocking down paralogs of acox-1 also contribute to increased PA14 survival in *rege-1(imm070)*. Fortunately, the *acox-1.5(tm15936)* mutant rescued the susceptibility of *rege-1(imm070)* to PA14, though partially, without shortening the survival by itself, providing evidence that the alteration of *acox-1.5* mRNA contributes to the decreased PA14 survival of *rege-1(imm070)*.
On the other hand, RNAi clone used for *ech-9* targets 58% of *ech-8*, a protein with 70% identity to *ech-9*. However, the third peroxisomal ECH paralog, *ech-4*, is not predicted to be targeted by the *ech-9* RNAi clone, but share close to 20 % identity to either *ech-8* or *ech-9*. Therefore, the little rescue we observed when knocking down *ech-9* in *rege-1(imm070)* could be due to either the RNAi efficiency being less than expected, or *ech-4* compensating for the functions of ech-8/ech-9 in the peroxisomal fatty acid beta-oxidation process. A detailed investigation is needed to understand the functions of the ECH paralogs.

Comparison of ETS-4 to PQM-1

9. The prevailing model regarding the regulation of gene expression by the IIS pathway is that DAF-16 functions as a transcriptional activator of Class 1 genes while PQM-1 upregulates Class 2 genes. Nuclear localization of the two transcription factors appears to be mutually exclusive such that in daf-2 animals where DAF-16 is constitutively active, PQM-1 remains in the cytosol, unable to access its transcriptional targets. Considering that ETS-4 also regulates Class 2 genes, how does it fit into this regulatory scheme?

The authors state that out of a total of 58 Class 2 genes that appear to be upregulated in an ETS-4-dependent manner, 49 are direct transcriptional targets of PQM-1. This may suggest that the two transcription factors co-regulate genes, perhaps in a cooperative manner, or that they are functionally redundant. Despite the apparent similarities between ETS-4 and PQM-1, there are indications from previous studies that they have distinct roles. For example, the lifespan of pqm-1 mutants is comparable to that of wildtype animals (Tepper et al., Cell 2013), but ets-4 mutants are long-lived (Thyagarajan et al., PLOS Genetics, 2010). Further, while pqm-1 is required for lifespan extension when IIS is inhibited, ets-4 is not. The authors have an opportunity in the current study to further distinguish ETS-4 from PQM-1 through experiments that directly compare them to each other. In particular, they should consider addressing the following questions:

• What is the total extent of overlap between genes that seem to be regulated by both ETS-4 and PQM-1? That is, in addition to Class 2 genes, how many other genes that the authors believe to be regulated by ETS-4 have been identified as direct PQM-1 transcriptional targets?

Thank you for your suggestion. During the revision period, we found that ETS-4 ChIP data is available in ENCODE. Thus, we extracted their data and performed a comparison. Among the 19,287 genes listed in the supplementary table from Tepper et al., Cell 2013, promoters from 712 genes (~3.7% of the total genome) are enriched by ETS-4 IP provided in Table S3. Additionally, 2,762 genes (~14.3% of the total genome) are enriched in PQM-1 ChIP data. We found that 401 genes are enriched by both ETS-4 and PQM-1 ChIP, which accounts for 56% of ETS-4 ChIP hits but only 14.5% of PQM-1 ChIP hits. This suggests that a subset of these two transcription factors' targets is indeed common, and there is a higher chance of *ets-4* targets also being PQM-1 targets than the other way around.

• Is the rege-1 pathogen susceptibility phenotype rescued by mutations in pqm-1 (or pqm-1 knockdown) as it is by mutations in ets-4?

Thank you for your suggestion. We have included pqm-1 mutant in both the PA14 survival assay and lifespan measurement. The data is presented in Figure 3B-C and Table S1. In summary, although ETS-4 and PQM-1 target an overlapping gene set, our results indicate that they are not functionally dependent. Specifically, missing pqm-1 does not affect ETS-4 function.

• Does a loss-of-function of pqm-1 rescue the shortened lifespan of rege-1 mutants?

Thank you for your question. We did measure the lifespan of both rege-1 single mutants and rege-1; pqm-1 double mutants, but we did not observe any differences between them, indicating that the introduction of the pqm-1 mutation did not affect lifespan. The results are presented in Figure 3C and Table S1.

• Is the nuclear localization of ETS-4 and DAF-16 mutually exclusive, as is the case for PQM-1 and DAF-16? An ets-4::gfp strain already exists (Habacher, Dev. Cell 2016), and the authors could examine its subcellular localization in presence and absence of DAF-16.

Thank you for your advice. Due to the time required for shipping and communication with the lab that had the *ets-4::gfp* integrated line, we were not able to obtain this data using the existing line. Therefore, we injected an extrachromosomal array containing *ets-4::gfp* into either wild-type or daf-16 mutant strains. Interestingly, we observed an increase in ETS-4::GFP nuclear localization in the *daf-16* mutant compared to the slight ETS-4::GFP nuclear localization found in wild-type, as shown in Figure 3D.

Data interpretation

10. In at least two cases the authors make statements in the Results section that appear to be at odds with what the data show. These instances are as follows:

1) In their description of the oil red O staining in Fig. 2D, the authors state that manipulation of either the IIS or TORC1 signaling pathways "is insufficient to rescue the fat loss phenotype of rege-1 (ht070)" (lines 207-208). However, the quantitation of staining in Fig. 2E shows that rege-1;daf-2 animals stained more intensely than both rege-1 and wildtype animals. The authors should reevaluate their assessment of the data and should also perform additional statistical tests to examine the differences between rege-1;daf-2 and rege-1 mutants in particular.

Thank you for your suggestions. We have reevaluated and modified our method for quantifying the oil-red staining to obtain more precise data. This has been helpful, as we initially believed that there was still a decrease in oil red staining in the *daf-2;rege-1* double mutant. However, after careful quantification, we found that the difference was statistically insignificant. Therefore, we conclude that the *daf-2* mutant is capable of fully rescuing the fat loss phenotype in the *rege-1* mutant.

2) In the Abstract, the authors claim that knocking down acox-1.5 "completely abrogated the survival difference between rege-1 mutants and wildtypes [sic]". This claim is repeated again in the Results section (lines 243-244) and Discussion section (lines 275- 276), but it is not what the data show. RNAi targeting acox-1.5 impairs immunity in both wildtype animals and rege-1 mutants. Combining the acox-1.5 knockdown with the rege-1 loss of function mutation reduces the survival of infected animals to the same extent as does acox-1.5 knockdown alone. It does not rescue the enhanced susceptibility to pathogen of rege-1 mutants or restore their survival back to wildtype levels. As written, the authors' description of the data is unclear and potentially misleading.

Thank you for your suggestion, and I am sorry that you think the statement was misleading. The mean survival time of mutants on PA14 varies for different reasons, such as *daf-2* resulting in longer survival. Thus, as defined in the revised manuscript, we use the lifespan in the mutant we tested (i.g. *daf-2* mutant) as 100% rescue and the lifespan in *rege-1(imm070)* as 0% rescue in our analysis. While we did observe a significant decrease in survival on PA14 in response to *acox-1* RNAi relative to wild-type, the magnitude of the decrease was so large that we were unable to calculate the percent of rescue, However, when we combined *acox-1* RNAi with *rege-1* mutant, we found that survival on PA14 did not decrease further, and even increased, compared to either *rege-1* mutant or *acox-1* RNAi on wild-type. These results suggest that the gene targeted by *acox-1.5* dsRNA

contributes to the decreases in survival caused by *rege-1* mutant. The survival assay performed in the new *acox-1.5* mutant supports our hypothesis, as shown in Figure 5.

11. While statistically significant, the magnitude of the difference in mean lifespan in rege-1 strains where ETS-4-regulated Class 2 genes is knocked down by RNAi is relatively low and is even lower in rege-1;ins-7 double mutants (Fig. 4C and D). Is there a threshold for a fold change in mean or maximum lifespan (or some other metric) that the authors established to determine whether or not a specific RNAi treatment had a substantial impact on the survival of rege-1 animals? Or was statistical significance the only criterion?
Thank you for your question. In the previous manuscript, statistical significance was the only criteria we used to determine rescue. However, in the revised manuscript, we have included percent rescue of mean lifespan as an additional way to quantify our data. As you pointed out, the differences are minimal in *ins-7* with other targeted gene knockdowns, so we have included only the PA14 survival curve for the *ins-7* with *ech-8/9* knockdown triple mutant in Figure S4.

Comments on Specific Figures

1. Fig. 1B shows that rege-1 expression is elevated in the rege-1(ht070) mutants as compared to wildtype. The authors may wish to point out that this makes the increased ets-4 expression in that strain even more remarkable and that it is a strong indication that the gene product of the ht070 allele has little to no ribonuclease activity.
   Thank you for your suggestion. We have included this observation as support for the defect of ribonuclease activity in *rege-1(imm070)*. Additionally, we have cited several previous papers, including our own, to support that a single amino acid substitution of aspartic acid to asparagine in the key catalytic residue is sufficient to abolish the ribonuclease activity.

2. Regarding Fig. 2:

• Fig. 2A should not be included as a figure panel but rather should be converted into a separate table.
Thank you for your suggestion, the figure 2A has now been changed to Tabel1.

• callout for Fig. 2B precedes the callout for Fig 2A in the text.
Thank you for noticing, we have modified accordingly.

• In the figure legend Panels B and C are not described in sufficient detail. The authors need to mention all genotypes tested.
Thank you for your suggestion. We have included all the genotypes in the figure legend.

3. In Fig. 4 panels C and D the color scheme in the symbol key should match that in the plot—specifically, the shade of green used to present RNAi-treated rege-1 mutants in the key is not the same shade as in the plots.
Thank you for bringing this to our attention. We will make sure to fix the error.

4. Regarding Fig S1:

• To avoid the first call-out in the text being to a supplemental figure (Fig. S1) could at least some of the data in that figure be moved to Fig. 1?

Thank you for your suggestion. The lifespan curves which were original in Figure S1 have now been moved to Figure 1B and 1D

• What statistical significance is indicated by the asterisks in the symbol key? (Mention p-value but no specific number)
Thanks for letting us know about this error. We apologize for any confusion caused and will correct it promptly.

5. The functional characterization of asp-12 seems to be out of place in Fig. S2, since the other panels in that figure pertain to read counts for TOR pathway components. Regarding the asp-12 knockdown data:
• The authors could consider including it as part of Fig. 4, but for the sake of completeness this would require additional experiments to knockdown asp-12 in wildtype and rege-1 mutants to generate the same type of data as shown for ech-9 and clec-52 in panel C.
Thank you for your inquiry. The knockdown of *asp-12* has been conducted in both wild-type and *rege-1(imm070)* strains and the results are presented in Figure 5 of our revised manuscript.
• Why was spp-3 the one ETS-4 regulated gene that was excluded from functional analyses?
As Class I genes were not identified as the targets in our previous analysis, only a few of the overlapped genes were selected for further analysis. Therefore, not all of the overlapped genes were tested.

6. Regarding Table S1:
• consider including average values from all replicates of a particular experiment to reflect the data that are depicted in corresponding figure panels
Thank you for your suggestion. We have included the average mean lifespan, mean lifespan relative to wild-type, and mean lifespan rescue by using the *rege-1* mutant as 0% rescue, and either wild-type or the background mutant as 100% rescue.

• consider breaking this up into separate supplemental tables to accompany specific figures instead of compiling all statistical data in one table. For example, the statistical data supporting Fig S1B and especially S1C (PA14 infection) could be moved up to appear immediately after the data pertaining to Fig. 1 so that the reader can directly compare the behavior of the two rege-1 mutant strains (tm2265 deletion strain and presumptive ribonuclease-dead ht070)
Thank you for your suggestion. The lifespan and PA14 survival curve of *rege-1(tm2265)* and *rege-1(imm070)* have all been moved to figure 1
• the statistical analysis of lifespan date should include p-values, as was provided for data pertaining to survival curves in Fig S1B and S1C but not for others.
Thank you for your suggestion. The p-value is now available for all possible combinations in Table S1

• the units of the values in the "mean lifespan" column are presumably days, but this should be specified. Also, is this truly mean lifespan or is it median lifespan (LT50)?
Thank you for your question. All the statistics for the survival curve and lifespan were calculated using the OASIS website (http://sbi.postech.ac.kr/oasis), and the mean lifespan was estimated by calculating the area under the survival curve using the following formula, $\hat{\mu}_\tau = \int_0^\tau \hat{S}(t)dt$ . Therefore, it represents the mean lifespan rather than the median lifespan..

## Comments on Writing

### Introduction

1. The introduction should be reorganized to first provide the reader with background information on post-transcriptional gene regulation and ribonucleases in particular.

Thank you for your comment. The introduction has been re-written. As there is limited knowledge about mRNA-specific ribonucleases, we have only briefly introduced the post-transcriptional regulation in the IIS and mTOR pathways.

2. The authors should add a paragraph discussing how mRNA degradation by ribonucleases allows animals to dynamically respond to their environment, in part by regulating metabolic pathways.

Thank you for your comment. Based on my knowledge, most ribonucleases that participate in regulating mRNA stability are typically part of the general RNA degradation machinery. However, Regnase-1 is a unique example of an endoribonuclease that can specifically target certain mRNAs. And this has been briefly included in the revised introduction.

3. Comment on the significance of the research question, as defined on lines 77-79.

Thank you for your comments. We have refined our main question to "how these two newly evolved metazoan proteins participate in *C. elegans* cellular processes and survival remains largely unknown. Specifically, whether an excess of ETS-4 results in detrimental effects in *C. elegans* and needs to be regulated by *rege-1* is still yet to be determined."

### Methods

4. More details pertaining to CRISPR gene editing methodology should be included. In particular, the authors should cite or describe the protocol that they followed and provide specific information regarding reagents, including guide RNA, repair templates, and the sequences of genotyping primers.

Thank you for your comments. We have provided a more detailed description of the CRISPR gene editing method used in our study, and all the oligos used have been listed in Supplementary Table 3.

5. Provide an explanation of the technique used to quantify GFP fluorescence intensity for the micrographs in Fig. 3C and for the quantification of oil red O staining in Figs. 2D and 4B. A description of statistical analyses should also be included.

Thank you for your suggestion. We have provided a detailed quantification approach for GFP intensity in the Pins-7::GFP, GFP::LGG-1, and ETS-4::GFP transgenes, as well as for the quantification of Oil Red O staining in the revised version of the Materials and Methods section. All statistical analyses were performed using Prism software and the specific statistical methods used are described in the figure legends.

6. Explain how animals were synchronized for RNAseq experiments and for oil red O staining experiments.

Thank you for your suggestion. We have updated the material and methods section to include the synchronization and growth protocols for both RNAseq and oil red O staining experiments.

### Discussion

7. Although it appears under the subheading of "Model," the first paragraph reiterates the summary of results that appears at the end of the Introduction section. It should be modified to describe the authors' model in Fig. 4E.
Thank you for your comment. We have modified the model section in the discussion.

8. The authors need to do significantly more to place their work in the context of other studies in the literature. At a minimum, they should specifically compare their findings to the following three papers:
1) Thyagarajan et al. PLOS Genetics 2010 (ETS-4)
2) Habacher et al, Dev. Cell 2016 (REGE-1)
3) Tepper et al., Cell 2013 (PQM-1)

Thank you for your comments, we have included "The role of DAF-16, PQM-1 and ETS-4 in modulating longevity genes" and "Role of ETS-4 in responding to environmental stress" in the Discussion.

9. Based on their GO term analyses of their DGE data, can the authors speculate as to why increased expression of ETS-4-regulated genes shortens lifespan and reduces immunity? Is there something in common to these genes that would make them particularly detrimental when expressed at high levels?

Thank you for your question. If we had analyzed the differentially expressed genes (DEGs) using GO terms, we would have reached a similar conclusion as Habacher et al. (2016) that immune and lipid catabolic genes are upregulated. However, we also observed that downregulated genes were enriched in immune-related genes, which required us to use a different analysis approach. By comparing our data with transcriptome analyses from various mutant lists, we were able to suggest that the DGE changes were likely related to the IIS and TORC1 pathways. Our epistasis studies further support this, indicating that the activation of the TORC1 signaling pathway was responsible for the shorter lifespan of the rege-1 mutant when exposed to PA14. Nevertheless, the reason why the activation of TORC1 signaling results in a decrease in pathogen defense remains unclear and requires further investigation. One possible explanation is that TORC1 activation reduces autophagy, which slows down the elimination of pathogens, resulting in decreased survival.

10. There is not a sufficient explanation of how fat and fat metabolism are related to immunity. This issue is pertinent to this study since the authors test for rescue of the rege-1 fat storage defect by inhibition of insulin signaling and TOR activity. The authors should comment on that topic in the Discussion and reference papers such as Anderson et al., PLOS Pathogens (2019).
Thank you for your suggestion. We have improved our quantification methods for the oil-red-O staining data to provide more accurate results. In addition, we have included a discussion on the possible contribution of the loss of fat to the decreased pathogen defense observed in rege-1 mutants. We appreciate your feedback and hope that these updates will improve the quality of our paper.

11. Consider addressing the evolutionary basis of a pathway (REGE-1-mediated mRNA degradation) that potentially came about in order to limit the host's response to infection (upregulation of ETS-4 transcriptional targets). How does this compare with other pathways that are induced by environmental stimuli? Are there similar regulatory mechanisms for responses unrelated to host defense in "lower" organisms?

Thank you for your question. As I mentioned in the revised introduction, both the Zc3h12a ribonuclease domain protein and ETS genes are only found in metazoans, meaning that no homologous protein in "lower" organisms can be used to describe their function. However, since RNA is considered one of the earliest forms of life and RNA regulatory networks are found prevalently in organisms that evolved prior to metazoans, it is likely that RNA played a significant role in pathogen defense mechanisms. One of the most well-known examples of this is the CRISPR system in bacteria, in which the Cas protein (either the ribonuclease or DNA nuclease) recognizes invading genetic material from bacteriophages and either destroys it or integrates a small part of it into the bacteria's own genome. In addition to regulatory RNase defense against pathogens, several regulatory RNAs are often used to regulate responses to environmental stimuli. For example, riboswitches can sense nutrients within cells and initiate gene expression in response, while sRNAs are commonly used to sense environmental conditions such as osmotic stress, as in the case of RybB.

Spelling, grammar, and formatting

12. There are many spelling and grammar errors throughout the manuscript. In addition, the authors need to be careful to use the proper C. elegans conventions for the formatting of gene and protein names.

Thank you for pointing that out. We will carefully examine the revised manuscript.

Lines 158 and 159: need citations for Class 1 and Class 2 genes as well as CHIP data for DAF-16 and PQM-1 referred to in the text

Thank you for bringing that to our attention. The missing references have been added.

Line 277: Why is there a callout to Fig. 4E when the sentence mentions a mouse study?

Thank you for your inquiry. The original intention of the paragraph was to establish a connection between different species, but we have since rewritten the model section.

Reviewer #2: Regen-1 is an immunity gene and knockout in mice has divergent outcomes depending on whether the knockout is whole-body or tissue-specific. In this study, Tsai et al. use C. elegans with mutated rege-1 (the sole ortholog of mammalian Regen-1) to test its role in survival and immunity. The authors find that the rege-1 mutant has decreased lifespan at baseline and decreased survival following exposure to Pseudomonas aeruginosa. Mutation of the rege-1 ribonuclease activity site alone is sufficient to phenocopy this decreased survival. rege-1 mutants have elevated levels of its known target mRNA, ets-4. RNA sequencing on rege-1 mutants and rege-1;ets-4 double mutants shows that ets-4-regulated genes are enriched for genes in the insulin/insulin-like signaling (IIS) and target of rapamycin complex I (TORC1) pathways. Crossing the rege-1 mutant to the daf-2 (IIS) or rsks-1 (TORC1) mutants improves survival and partially rescues the fat loss phenotype of the rege-1 mutant. Tsai et al. also test the effect of crossing the rege-1 mutant to ins-7 and acox-1.5 (targets of ets-4) mutants and saw mild improvements in survival. Overall, the authors conclude that in a rege-1 mutant, excess ets-4 upregulates IIS and TORC1 signaling, leading to poor survival. This study adds contributes to our

knowledge of how an understudied gene, rege-1, is involved in survival and immunity and highlights the role of the nutrient metabolism pathways IIS and TORC1 in mediating rege-1 survival. However currently there are some concerns over data analysis supporting the key findings, statistics and replicates that leave this work short of being publication ready. Key Issues to be Improved:

• A key finding of the paper from the RNA seq is the changes in PQM related genes. More data is needed to flesh out this finding – is PQM localization changed v DAF-16? Does modulating PQM impact the effects of rege-1 or the daf-2 'rescue'?

Thank you for your question. It is indeed important to address. Due to time constraints during revision, we conducted one analysis and two experiments in the revised version to address some of your questions:

1. Up-regulated genes in rege-1 mutants are significantly enriched for genes targeted by both ETS-4 and PQM-1, as revealed by ChIP analysis from existing data (Table S3).
2. The pqm-1 mutation does not rescue the poor PA14 survival or shorter lifespan of the rege-1 mutant (Figure 3B-C and Table S1).
3. In wild-type conditions, DAF-16 hinders the nuclear localization of ETS-4 (Figure 3D).

• Effects of mTORC1: Is activity of mTORC1 altered in rege-1 mutants? This could be examined using phos S6K/RSKS-1 antibodies used elsewhere in C. elegans studies

Thank you for your suggestion. We did borrow some phos S6K/RSKS-1 antibodies from a neighboring lab to try them out. Although they initially seemed to work, we later experienced inconsistent results. While we continued troubleshooting, due to the time constraints for the revision period, we opted to replace our western data with two other sets of data.

1.) Epistatic regulation of another TORC1 pathway gene. Here we have also included an rsks-1 mutant to show that both TORC1 mutants (raga-1 and rsks-1 mutant) result in fully rescues of poor PA14 survival of rege-1 mutant (Figure 4A-B)
2.) The inhibition of autophagosome formation in rege-1 mutant. Activation of TORC1 is known to prevent the initiation of autophagy by phosphorylating ULK-1. Therefore, we used the decrease in autophagosome formation in the rege-1 mutant as an indication of mTOR activation (Figure 4C).

• The authors should provide more details about mutants used in the study to assist with proper interpretation of the results. How much of the rege-1(tm2265) coding sequence is deleted? Is it a total null or is there functional protein remaining? Is the ribonuclease activity site that is mutated in rege-1(ht070) also mutated in the tm2265 allele?. Verify that the rege-1(ht070) mutation abolishes ribonuclease activity – noy citation is given and the fig cited does not carry out those exps. Give clarifying details about the ets-4(ok165) mutant. Is it a deletion? Where and how much of the gene is deleted? Same for the ins-7 mutant used.

Thank you for your suggestions. We have included a detailed description of all the deletions used in this study in the revised version of the paper, with proper citations. Additionally, we would like to inform you that, due to some miscommunications with wormbase, the allele names from our lab are now initiated with "imm".

• Some figures have missing statistics, replicates, or information about how many replicates weree of poor PA14 survival phenotype of rege-1(imm070) performed: No replicate information for the figures in S1. No statistics in S1D or S1E. S1B and S1C only have 1 replicate.
Thank you for pointing out these careless mistakes. We have now included all the replicates for the survival curves, and this revised information is now included in Table S1.

• The authors should check that all experiments have clearly explained methods either in the Methods section and/or in the figure legend. For example, for figure S1E:
o No information on the methods of the imaging performed. For example how old were the worms and are they synchronized?
We appreciate your question and have updated the materials and methods section to include information on the developmental stage of the worms used in the study.

o The worms in the far right bottom panel of S1E look morphologically distinct from the ones above (which are supposedly the same genotype).
Thank you for your suggestion. We have retaken the images and replaced Figure S1 in the revised version of the manuscript.

o Total RFP in the intestine is used as a proxy for bacterial clearance. How does total amount of RFP in intestine tell you anything about clearance? For this it would require an image while they feed, then remove them from the RFP-PA14 lawn for a set amount of time and then image again? Can the authors provide more details about how this experiment was performed in the Methods section?
We appreciate your suggestion. To provide more clarity, we have added a section titled "PA14 clearance and avoidance assay" in the material and methods that provides a detailed description of the experiments.

• Although in figure 4 rege-1 loss indeed does not suppress lifespan in the presence of acox-1.5 RNAi the result is more complicated than reported in the text and needs further elaboration. There is an interesting contextual effect of acox-1.5 RNAi in that it shortens the lifespan of WT animals but increases the lifespan of rege-1 mutants. This shd be tested statistically and discussed in results.
Thank you for your suggestion. In the revised manuscript, we have included the acox-1.5 mutant to perform the PA14 survival assay, and the results are much clearer than those obtained with RNAi. Specifically, we observed no decrease in survival in the acox-1.5 mutant, but a 60% rescue in the rege-1;acox-1.5 double mutant. While the difference between wild-type and rege-1 mutant is more distinct when incorporating the acox-1.5 mutant rather than feeding with acox-1.5 dsRNA, the rescue is now clear. We also discuss the impact of peroxisomal fatty acid beta-oxidation on rege-1 mutant phenotypes in the Discussion section.

Minor Points
• In fig 4C stats in WT v WT+ RNAi need to be included
Thank you for your suggestion, the figure legend has been re-written
• Line 64 – “Pseudomonas” spelled incorrectly

Thank you for pointing out the mistake, we have corrected it.

• Line 107 – An additional introductory sentence regarding what's known about ets-4 would improve this section. What's known about it? Is it a transcription factor? What genes is it known to regulate?
Thank you for your question. We have updated our introduction to include the background of *ets-4*
• Line 153 – How were the results from WormExp v2.0 verified and why do the numbers differ after verification? Add details in Methods section.
Thank you for the suggestion. Since we were not sure of how the website calculate the overlapped genes (we sometimes saw more number of overlapped genes than the genes we provide), we decided to remove the overlapped genes in the revised version of manuscript.

• Line 155 – When comparing the overlapping genes, the denominators are 41 and 17. Where do those numbers come from? They aren't in the Venn Diagram. Many comparisons are being made from the same RNA sequencing experiment and clarifying how the genes are begin subset would help the reader better interpret the results.
Thank you for your comments, and we apologize for the confusion. We have now included the number of differentially expressed genes in Figure S2A and the number of individual clusters next to Figure 2A.

• Line 186 – If the RNA sequencing changes were similar to the transcriptional profile of a rsks-1 mutant, why cross to a raga-1 mutant? Some justification for this choice in the text would clarify for the reader
Thank you for your question. We have included the PA14 survival experiment in *rsks-1(tm1714)* in the revised manuscript. Although we requested the same rsks-1 mutant strain from lab in Taiwan to test the hypothesis before the previous manuscript, the strain we obtained was contaminated with wild-type worms. Due to the pandemic back then and the shipping from USA was significantly delayed, so we decided not to request the same strain from CGC, which is why we were unable to include the data in the first place. The *rsks-1(tm1714)* mutant used in the revised manuscript was obtained from the National BioResource Project (NBRP) in Japan, which save us some time in shipping and also rule out the potential effect from a specific mutant strain background.

• Line 269 - a more direct test of whether TOR pathway activity is increased would be to do Westerns for phosphorylation of TOR or its downstream targets
Thank you for your suggestion, as mentioned previously, since the antibody for S6K are made for human and mice, even though we did give it a try, the results were not as consistent as we would expect. Thus, we can not provide the western data at this point.

• Line 293 – needs citation

Thank you for bringing this to our attention. We have added the appropriate citation.
