# Supplementary material for: Rege-1 promotes C. elegans survival by modulating IIS and TOR pathways
Source: PLoS Genet. 2023 Aug 9;19(8):e1010869. doi: 10.1371/journal.pgen.1010869 (PMC10441803; doi:10.1371/journal.pgen.1010869)
Supplement: Attachment — Submitted filename: Tsai revisions comments_final.pdf [file pgen.1010869.s013.pdf]

In my comments on the authors' original manuscript, I identified four areas of major concern, pertaining to the RNASeq data; the strength of the connection between *acox-1.5*, *ech-9* and the TOR pathway; functional differences between ETS-4 and PQM-1; and the interpretation of data from Fig. 2 (oil red O staining) and Fig. 4 (relative lifespan of mutant strains). In the revised manuscript, Tsai and colleagues have addressed each of these, adding important new data and analyses that help to strengthen many of their claims. They have also rearranged the figures, in part in response to some of my comments, but also to group related datasets that were scattered between different figures and the supplemental information in the previous draft. This change has substantially improved the organization of the paper as a whole. Finally, the authors have systematically addressed editorial comments that I made regarding their figures and their writing. Although many grammatical and typographical errors remain and need to be addressed, I am satisfied with how the authors have responded to my comments, for the reasons that I articulate below. Overall, the changes that the authors have made significantly enhance the quality and potential impact of the manuscript.

Differential Gene Expression (DGE) studies

1) Volcano plots and principle component analyses (PCA) have been added in Fig. S2B, making the authors' statistical analyses of their data more clear. In particular, the fold difference in expression among the Class 2 genes of interest between the different genotypes tested is now apparent, as is the statistical significance of those changes. The PCA shows that there is a reasonable degree of similarity between biological replicates included in the DGE experiments. This is particularly important in light of point (2) below.

2) Since there was some inconsistency in the gene expression data between two replicates of the *P. aeruginosa*-fed *ets-4* animals, the authors have removed these samples from their DGE analyses, in direct response to my comment. The analysis now compares expression in wildtype, *rege-1* and *rege-1;ets-4* animals (Fig. 2A). It is therefore still possible for the authors to identify putative ETS-4 transcriptional targets based on the criteria that when compared to their expression in wild type, the expression of those genes should be increased or decreased in *rege-1* mutants (because of increased ETS-4 activity) but not in *rege-1;ets-4* animals (Table S2). Although it would be reassuring to see reduced expression of genes that are positively regulated by ETS-4 in *ets-4* mutants when compared to their levels in wildtype animals, without these data the authors' analysis of publicly available ChIP-seq data from ENCODE, which is a new addition to this version of the manuscript, provide a reasonable second line of evidence to identify genes regulated by ETS-4 (Fig. 2B).

3) Responding to my comment, the authors rearranged the gene expression data in the heatmap (Fig. 2A) to group genes within six categories, according to their relative expression level in *rege-1* mutants in the absence or presence of a bacterial pathogen (*P. aeruginosa*). The new organizational scheme of the heatmap reveals how expression patterns of putative ETS-4 targets change in response to infection. However, it is a departure from the hierarchical clustering that is typically used to construct heatmaps from DGE data (which is how the authors originally presented the heatmap in the original manuscript), and so some readers may object to it. My comment asked Tsai and colleagues to "more clearly describe the categories of genes potentially regulated by ETS-4 that are revealed in their DGE analyses," and my intention was for them to accomplish this in prose instead of reconfiguring the heatmap. This may yet be the best option.

Connection between ETS-4 and the TOR pathway

1) Fig. 4 in the revised manuscript is entirely devoted to presenting data supporting the possibility that *rege-1* intersects with the TOR pathway. Importantly, the authors added two new pieces of data that did not appear in the original version of the manuscript. First, characterization of *rsks-1(tm1714);rege-1(imm070)* double mutants in the *P. aeruginosa* infection assay (Fig. 4B) shows that the survival of these animals is similar to *raga-1(ok386);rege-1(imm070)* mutants (Fig. 4A), allowing the authors to conclude that eliminating two different *bona fide* components of the TOR pathway rescues the susceptibility phenotype of *rege-1* mutants. Since ETS-4 is more transcriptionally active in *rege-1* mutants (as the authors show in Figs. 2 and 5A), these data help to strengthen the authors' claim that ETS-4 influences the TOR pathway in *C. elegans*. Further substantiating this assertion are the results of a second, new fluorescence-based assay that is a more direct readout of TOR activity. Specifically, the authors use the *gfp::lgg-1* reporter to demonstrate that autophagy is inhibited in *rege-1(imm070)* mutants (Fig. 4C), as is the case when the TOR pathway is activated. Taken together, the new data in Fig. 4 in the revised manuscript help to more firmly establish a possible connection between *rege-1* and the TOR pathway, strongly implying a role for ETS-4 in regulating TOR.

2) Through their own RNAseq (Fig. S2) and qRT-PCR studies (Fig. 5A) along with analyses of ENCODE ChIP-seq data (Fig. 2B), the authors show that *acox-1.5*, encoding a peroxisome beta oxidation enzyme, is a transcriptional target of ETS-4. Studies of the mammalian ortholog of *acox-1.5* indicate that acetyl coA produced during fatty acid oxidation activates TOR. Therefore, *acox-1.5* could directly link ETS-4/REGE-1 to the TOR pathway in *C. elegans*. I asked the authors to provide greater evidence for this possibility in the revised version of the manuscript, and in my opinion the most compelling evidence comes from their new studies with *acox-1.5(tm15936)* mutants. Data from experiments with this mutant represent a key addition to the paper because they resolve potential ambiguity in genetic interaction studies where *acox-1.5* expression was knocked down by RNAi for reasons that the authors carefully discuss in the Results section. By comparison to RNAi treatments (Fig. 5G), it is much more apparent that *acox-1.5* loss of function partially rescues the *rege-1* enhanced susceptibility to pathogen phenotype in *acox-1.5(tm15936);rege-1(imm070)* mutants (Fig. 5H). This bolsters the authors' argument that the *rege-1* phenotype is due in part to the enzymatic activity of ACOX-1.5. The authors make further use of the *acox-1.5(tm15936)* to yield more conclusive results to support this possibility by measuring fat content in *C. elegans* through oil red O staining. Whereas RNAi knockdown of *acox-1.5* did not significantly change the amount of fat in *rege-1(imm070)* mutants (Fig. 6B), new data show that the *acox-1.5(tm15936)* partially rescues the *rege-1* fat loss phenotype, according to the authors' revised method of quantifying oil red O staining (Fig. 6C). This is a critical result because it suggests that ACOX-1.5 activity may be increased in *rege-1* mutants, an important component of the authors' model (Fig. 7), which is not implied by the results of RNAi experiments. Characterization of the *acox-1.5(tm15936)* mutants therefore provides the authors with data that are necessary to substantiate some of the claims made in the original manuscript regarding the mechanistic basis for the *rege-1* phenotypes that were based on less-convincing evidence. In aggregate these data suggest that ACOX-1.5 indeed influences fat metabolism in *C. elegans* and that increased ACOX-1.5 enzymatic activity may underlie the impaired immunity of *rege-1* mutants which appears to be entirely attributable to activation of TOR, as described above. Thus the authors' data point to regulation of TOR through beta oxidation in *C. elegans*, analogous to the scenario in mammals.

Comparison of ETS-4 to PQM-1

Considering that the Tsai and coworkers found that 49 of 58 Class 2 genes that appear to be upregulated in an ETS-4-dependent manner are direct transcriptional targets of PQM-1, I asked them to address a series of questions that would allow them to functionally distinguish ETS-4 from PQM-1. These questions were addressed by the authors in at least three new experiments whose results are reported in Fig 3. Having shown in Fig. 1 that *ets-4* loss-of-function mutations rescue the reduced lifespan of *rege-1* mutants both in the presence and absence of bacterial pathogens, complementary studies indicate that mutations affecting *pqm-1* are incapable of such rescue (Figs. 3B and C). These data suggest that while *ets-4* appears to be regulated by REGE-1, *pqm-1* does not. Further characterization of ETS-4 using a new transgenic ETS-4::GFP line constructed by the authors indicated that, similar to PQM-1, the nuclear localization of ETS-4 increases in the absence of DAF-16 (Fig. 3D). This implies that ETS-4 and PQM-1 may be present in the nucleus at the same time and could co-regulate some genes. The authors' comparison of ETS-4 and PQM-1 ChIP data in ENCODE (Table S3) indicate that there is, in fact, some overlap between the genes regulated by the two transcription factors. These experiments and analyses add value to the paper because they highlight similarities and differences between ETS-4 and PQM-1 and because they shed new light on the means by which Class 2 genes are regulated. Not only does this enhance the level of mechanistic detail of the authors' model (Fig. 7), but it informs readers who may be particularly interested in the regulation of genes that lie downstream of the insulin signaling pathway, including lifespan determinants.

Data Interpretation

I raised three concerns regarding the authors' interpretation of their data, each of which have been addressed in the revised manuscript. In particular:

- The authors modified their method for quantifying oil red O staining (which applies to all of the data presented in Fig. 6). This led them to revise their conclusion regarding the ability of *daf-2* mutations to rescue the fat loss phenotype of rege-1 animals.

- Characterization of the *acox-1.5(tm15936)* mutant clarified conclusions regarding genetic interactions between *acox-1.5* and *rege-1*, as described above.

- The authors have included percent rescue of mean lifespan as an additional way to quantify survival data from lifespan and infection assays. This helps to clarify whether particular knockdowns substantially impact survival in the *rege-1(imm070)* background, especially when targeting the Class 2 genes in Fig. 5.

Comments on Specific Figures

The authors have addressed all comments that I made on specific figures. Included in their revisions in this version of the manuscript is an improved and more complete statistical analysis of the survival data from their infection assays (Table S1).

Comments on Writing

All of the comments that I made regarding the Introduction and Methods have been addressed satisfactorily.

The authors have made significant changes to the Discussion, partitioned by six subheadings, in part in response to issues and questions that I raised and also to reflect new data that they report in the revised version of the manuscript. While the points considered by the authors in the Discussion are reasonable, I believe that the flow of this section could be dramatically improved by reordering the topics, according to the outline in the table below.

Current order	Suggested new order
1) Model	1) Model
2) Role of ETS-4…	2) Role of ETS-4…
3) Does loss of fat…	3) Role of DAF-16, PQM-1…
4) Role of DAF-16, PQM-1…	4) The impact of fatty acid…
5) REGE-1 regulation of TORC1…	5) Does loss of fat…
6) The impact of fatty acid…	6) REGE-1 regulation of TORC1…

Spelling, Grammar, and Formatting

There are still many spelling, grammatical, formatting, and typographical errors throughout this version of the manuscript, both in the text and in the figures. A few examples are as follows:

- Introduction, page 104: "associated Argonautes, have been found regulate metabolic pathways" (should be: have been found *to regulate*)

- Introduction, page 105: "exon regeneration" (should be *axon* regeneration)

- Discussion subheading: "Role of ETS-4 in respond to environmental stress" (should be: *response* to environmental stress)

- The empty RNAi vector L4440 is frequently improperly referred to as L440, especially in Fig. 5.

These issues must be addressed in full before the manuscript can be considered to be suitable for publication.
